# Risk factors and long-term outcome of disease extent progression in Asian patients with ulcerative colitis: a retrospective cohort study

**DOI:** 10.1186/s12876-018-0928-2

**Published:** 2019-01-10

**Authors:** Yun Qiu, Baili Chen, Yufei Li, Shanshan Xiong, Shenghong Zhang, Yao He, Zhirong Zeng, Shomron Ben-Horin, Minhu Chen, Ren Mao

**Affiliations:** 1 Department of Gastroenterology, The First Affiliated Hospital of Sun Yat-sen University, 58 Zhongshan II Road, Guangzhou, 510080 People’s Republic of China; 2 Department of Gastroenterology, Hepatology and Nutrition, Digestive Diseases and Surgery Institute, Cleveland Clinic, Cleveland, USA; 3 Department of Gastroenterology, Sheba Medical Center & Sackler School of Medicine, Tel-Aviv University, Tel Aviv, Israel

**Keywords:** Disease extension, Progression, Ulcerative colitis, Risk factor

## Abstract

**Background:**

The incidence of Ulcerative colitis (UC) in Asia is increasing but reports on its long-term course are few. The aim of this study was to identify risk factors predictive of extent progression in Asian patients with UC and to evaluate the clinical outcome by longitudinal follow-up.

**Methods:**

We retrospectively analyzed 518 UC patients without ascending colon involvement at diagnosis who were regularly followed and underwent colonoscopy between 2003 and 2016 in an Asian tertiary referral center. Proximal disease extension and associated risk factors were analyzed.

**Results:**

A total of 91 (17.6%) patients experienced proximal disease extension followed for a median period of 7.5 years. The median time for extent extension was 16.1 months (interquartile range (IQR) 8.3–42.2). The cumulative rate of disease extension was 9.9, 14.9, 19.6, 24.6 and 30.5% at 1,2,3,4 and 5 years after diagnosis. 43 (12.0%) patients with E1/E2 progressed to E3, and 40 (21.9%) with E1 progressed to E2. Of patients diagnosed with E3 involvement limited to the hepatic flexure distally, 8 (13.3%) progressed to pancolitis. Cox regression analysis found that disease extent at diagnosis was the sole predictor of disease extension (odds ratio (OR),1.74, 95% confidence interval (CI) 1.18–2.57, *p* = 0.01). Proximal disease extension was associated with disease relapse (*p* = 0.03) and increased use of steroids and immunosuppressive agents (*p* <  0.01).

**Conclusion:**

UC is a dynamic disease and that the disease extension in Asians was comparable to that in Caucasians. Proximal disease extension increased the risk of disease flare and treatment intensification.

## Background

Ulcerative colitis (UC) is a dynamic disease that can progress to involve increasing segments of the colon over time [1]. In population-based studies, around one-third of UC patients with limited disease at diagnosis will have proximal disease extension within 10 years [2]. As one of the major determinants of long-term disease course, proximal disease extension is associated with a more aggressive disease course evident by higher rate of therapeutic requirements and colectomy [3], when compared with those presented extensive colitis at diagnosis [4]. Till now, the indentified risk factors included younger age, extra-intestinal manifestations, refractory disease at diagnosis and non-smoking, [3, 5, 6] but these factors varied between studies which merits further study.

Studies on the changes in the disease course over time of Asian patients with UC are still lacking giving the rising incidence of UC in Asia [7]. According to previous studies, phenotypic differences exist between Asian patients with UC and Caucasians [7–9]. However, the specific way of disease extent progression in Asian UC populations has been poorly described with limited sample size and short-term follow-up [10–12]. It remains unclear whether Asian patients with UC present as a different way of disease extent progression from Caucasians.

The aim of this study was to evaluate the dynamic disease evolution of a large cohort of UC patients from an Asian tertiary center. We also assessed risk factors and long-term outcomes of patients with disease extension.

## Methods

### Study design

This was a retrospective cohort study of patients with an established diagnosis of UC in the Gastroenterology Department of the First Affiliated Hospital, Sun Yat-sen University (China). We retrieved detailed demographic and clinical information from an Inflammatory Bowel Disease (IBD) registry, which has been prospectively maintained since 2003. The information obtained from the registry included gender, age, date of symptom onset, date of diagnosis, smoking status, disease activity, disease extent at diagnosis and during the course, medication use, and date of colectomy during follow-up. Initial disease activity in the cohort was evaluated using Truelove and Witts’ criteria [13]. The diagnosis of UC was based on a combination of medical history, clinical evaluation, and typical endoscopic and histological findings [14].

### Definitions

The extent of disease in UC was defined according to the Montreal classification as E1: proctitis (proximal extent to the sigmoid colon), E2: left-sided (to the splenic flexure) or E3: extensive disease (beyond the splenic flexure). Proctitis was defined as disease < 15 cm from the anal verge. Disease extension was defined as a proximal progression endoscopically from the initial extent at diagnosis. Specifically, disease extension was defined limited UC [E1 or E2] at diagnosis with progression to E2 or extensive colitis [E3]. For patients diagnosed with E3 involvement limited to the hepatic flexure distally, disease extension was defined as progression to pancolitis (proximal to hepatic flexure). In cases without investigative colonoscopy procedures before surgery owing to acute complications, disease extent evaluation was based on macroscopic description of the surgical specimen [15]. Smoking habit was recorded at inclusion. Patients were defined as smokers if they consumed at least 7 cigarettes/week and non-smokers if they had never smoked or ceased before diagnosis [16]. Diagnostic delay was defined as the time interval between the onset of symptoms until diagnosis.

### Treatment policy

The treatment regimen in UC during the follow-up period was based on a step-up approach [17]. Briefly, 5-aminosalicylates (5-ASA) (topical and/or oral) was used for the treatment of non-severe flare-ups, maintenance and first-line prophylaxis. Corticosteroid therapy is used in patients with moderately to severely active UC. Thiopurines or anti-tumor necrosis factor (anti-TNF) agents are used in steroid-dependent or steroid-refractory patients. For hospitalized patients with acute severe UC, a rescue medical therapy with anti-TNF agents or intravenous cyclosporine or colectomy is considered in patients refractory to intensive steroids treatment.

Our patients are followed up every 1–3 months at the outpatient clinic at regular intervals according to their conditions. Of note, the second and subsequent endoscopic assessments were usually planned within 6–12 months intervals by the treating physician to assess the response to therapy. All endoscopic procedures were performed by skilled endoscopists (BLC and YH). Endoscopic severity was evaluated with the Mayo Endoscopic Score [14].

### Ethics, consent and permissions

This study protocol (IRB number: 2015–47) was approved by the Clinical Research Ethics Committee of The First Affiliated Hospital of Sun Yat-sen University. There was a waiver of consent in the present retrospective study as this project meets the criteria according to Health & Human Services regulations (45 CFR 46).

### Statistical analysis

The statistical analyses were performed with SPSS (version 19) statistical software (SPSS Inc., Chicago, IL). The data are given as numbers and percentages or medians and interquartile range (IQR). Comparisons between means were performed using the t- test for independent samples. Categorical variables were compared using the χ^2^ test and the Fisher’s exact test. Univariable Cox regression was used to identify candidate predictors for inclusion in the multivariable model. A criterion of *p* ≤ 0.10 was used to identify candidate predictors which were further fitted by a forward selection procedure to eliminate non-significant variables. The odds ratios [OR] derived from the Cox’s models are presented with 95% confidence intervals [CIs]. Survival analysis was performed using Kaplan-Meier analysis based on log-rank test. Statistical significance was set at *p* <  0.05.

## Results

A total of 632 patients fulfilled the diagnostic criteria for UC and were included in the study. The median follow-up time was 7.5 years (IQR 5.6–8.1 years). Baseline characteristics are shown in Table 1.

**Table 1:** Characteristics of Patients

	N	%
Gender, n [%]		
Male	371	58.7
Female	261	41.3
Age [years] at diagnosis, median [IQR]	37.02(27.49–46.88)	
Smoking		
Never smoked	513	81.1
Disease extent at diagnosis, n [%]		
E1	183	34.4
E2	175	32.9
E3	174	32.7
Severity		
mild	348	66.6
moderate	145	27.7
severe	30	5.7
Medical treatment during follow-up		
5-Aminosalocylates^a^	373	59
oral	196	31
topical	56	8.9
oral and topical	121	19.1
Corticosteroid	224	35.4
Immunomodulator	109	17.3
AZA	99	15.9
6-MP	11	1.8
Methotrexate	18	2.9
Thalidomide	5	0.8
Cyclosporine	7	1.1
Biologics	11	1.7

Note: ^a^ Patients with combined use of 5-ASA and steroid/IMMs were counted in the Steroid/IMMs group

### Disease extent during follow-up

**Fig. 1:**
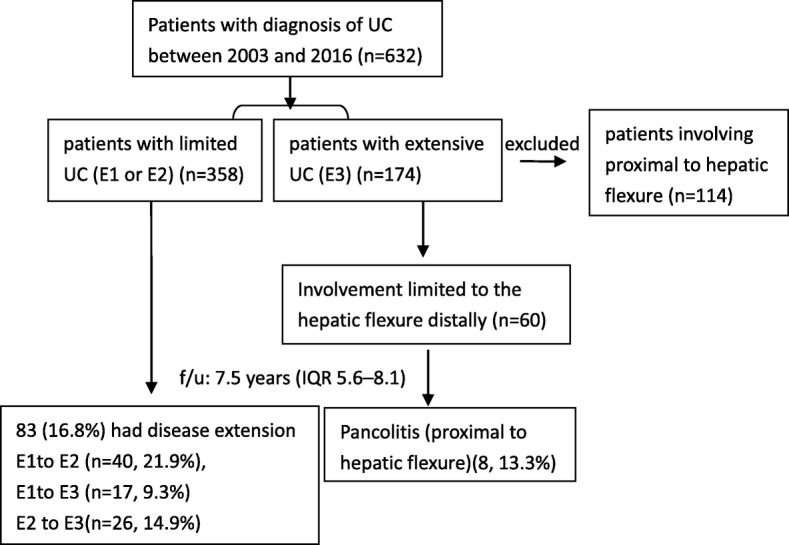
Flow chart of the study

Of the 632 patients with UC, 458 (72.5%) patients had limited UC (E1 or E2), whereas 174 patients were diagnosed with E3 at the time of diagnosis, including 114 patients with involvement proximal to hepatic flexure (pancolitis), and 60 patients with involvement limited to the hepatic flexure distally. One hundred and seventeen (18.5%) patients had skipped periappendiceal lesions. Therefore, we identified risk factors for disease extension and subsequent outcome in 518 cases, after excluding the 114 patients with pancolitis (Fig. 1).

**Fig. 2:**
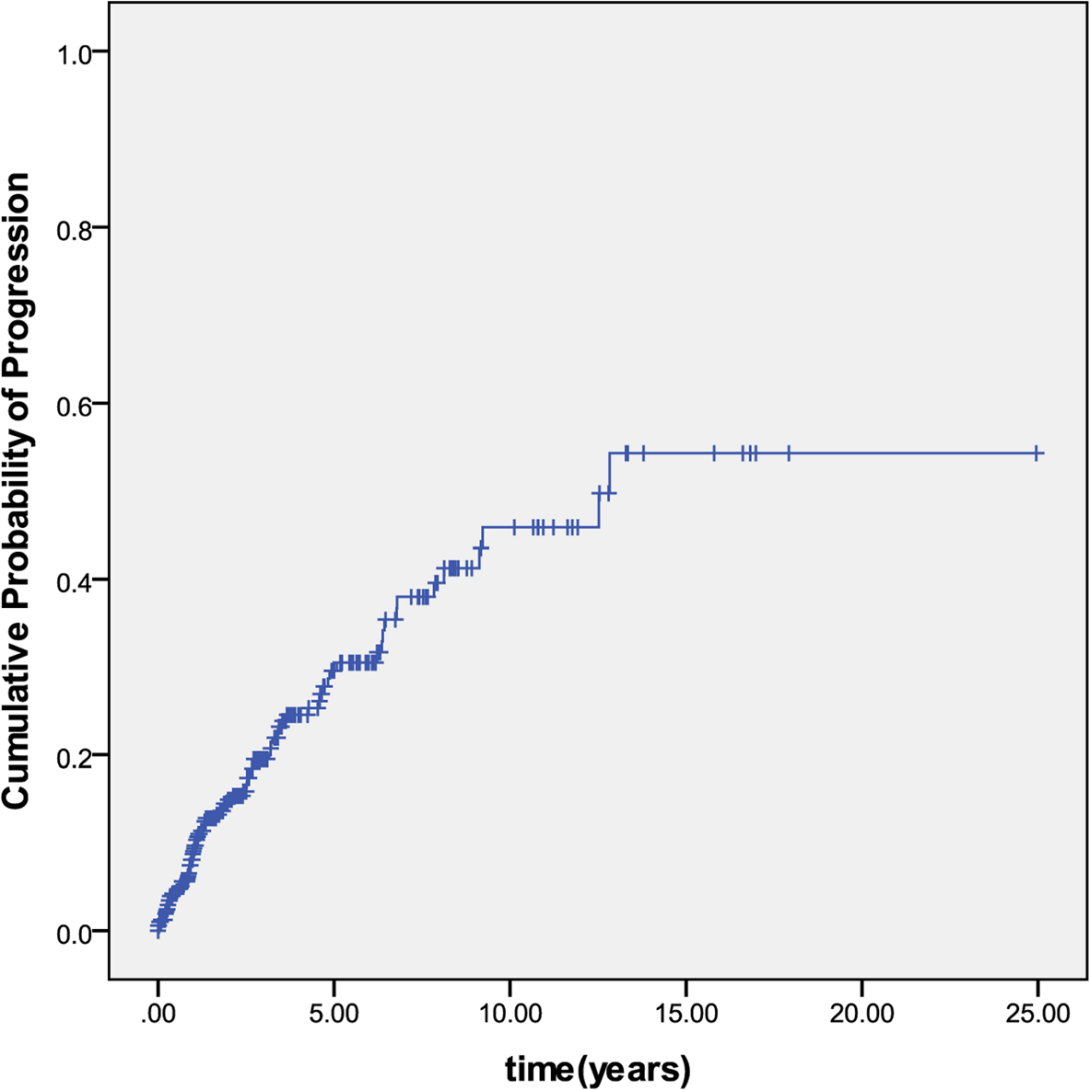
The cumulative rate of disease progression in patients

Of the 518 patients, 91 (17.6%) had disease extension during the follow-up. The median time for any progression of disease extent was 16.1 months (IQR: 8.3–42.2 months). The cumulative rate of disease extension was 9.9, 14.9, 19.6, 24.6 and 30.5% at 1,2,3,4 and 5 years after diagnosis (Fig. 2). More specifically, during the follow-up period of the patients diagnosed with E1, 40 (21.9%) progressed to E2 and 17 (9.3%) progressed to E3. Of patients diagnosed with E2, 26 (14.9%) progressed to E3. Of patients diagnosed with E3 involvement limited to the hepatic flexure distally, 8 (13.3%) progressed to pancolitis (proximal to hepatic flexure) (Fig. 1). Overall, 43 (12%) patients with E1/E2 progressed to E3, of whom 41 (11.5%) patients experienced disease progression within the first 5 years after diagnosis.

**Fig. 3:**
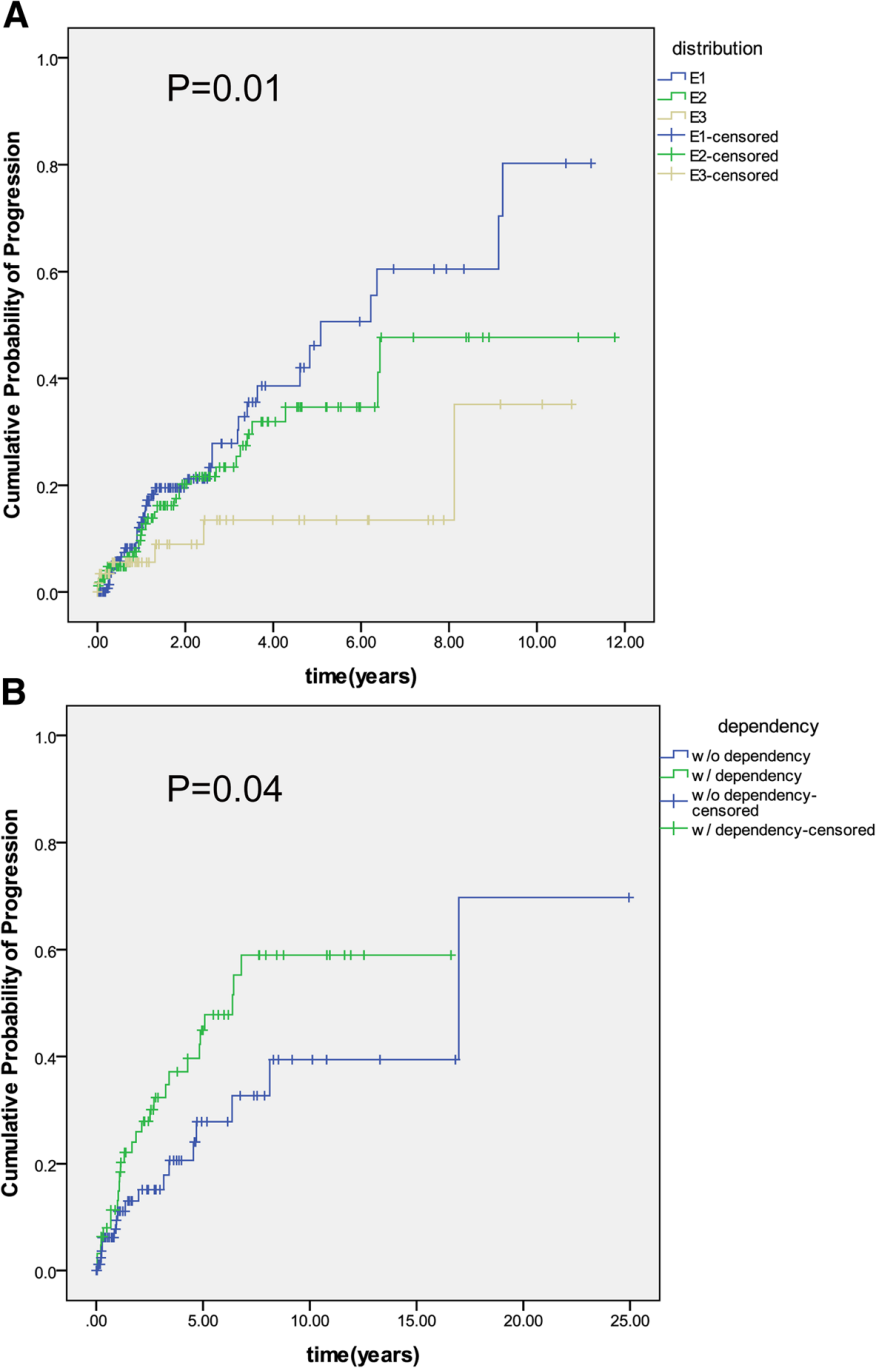
Comparison of disease progression in patients with **a**) different disease extent at diagnosis and **b**) steroid-dependence or not. The effect of disease distribution or steroids dependence on the probability of disease progression was evaluated using time-to-event [survival] methods for censored observations, because of the varying length of follow-up. Time to event was calculated from the date of diagnosis to the date of disease progression or censoring. Kaplan–Meier estimates were used to draw the cumulative incidence curves, compared by log-rank tests

For patients with initial E1, the proportion of disease progression at 1, 5 and 10 years after diagnosis were 13.0, 46.1 and 80.8% respectively, which was significantly higher than patient with E2 (E2, 11.7, 34.6 and 48.2%) and E3(E3, 5.6, 13.5 and 36.3%)(*p* = 0.01, Fig. 3a).

The cumulative rates of disease progression were 13.1, 44.9 and 59.4% at 1, 5 and 10 years after diagnosis in patients with steroid-dependence, which was significantly higher than patients without steroid-dependence (11.1, 27.9 and 39.4%, *p* = 0.04) (Fig. 3b). A Cox regression model was constructed to identify predictive factors for disease extension. As shown in Table 2**,** after including all variables found to be associated with the primary outcome on univariate analysis with *p* value of < 0.1, a multi-variable analysis demonstrated that disease extent at diagnosis was the sole significant predictor of disease progression (OR = 1.74, 95% confidence interval (CI) 1.18–2.57, p = 0.01).

**Table 2:** Risk factors predictive of disease extension in UC

	Univariate analysis			Multivariate analysis		
	OR	95.0% CI	*p*	OR	95.0% CI	*p*
Gender						
female	1.1	0.61–1.97	0.76			
male	Reference					
Age						
< 40 yrs.	1.57	0.79–3.12	0.20			
> 40 yrs	Reference					
Smoker						
yes	0.47	0.15–1.47	0.17			
no	Reference					
Extent						
Limited(E1/2)	1.99	1.29–3.07	< 0.01	1.74	1.18–2.57	0.01
extent	Reference					
Disease severity						
severe vs	1.1	0.58–2.10	0.77			
mild	Reference					
Corticosteroids use						
yes	0.64	0.32–1.27	0.20			
no	Reference					
Immunosuppressive agents use						
yes	0.47	0.24–0.91	0.02			
no	Reference					
Diagnostic delay						
< 6 months	1.04	0.60–1.80	0.89			
> 6 months	Reference					

### Outcome of patients with disease progression

**Fig. 4:**
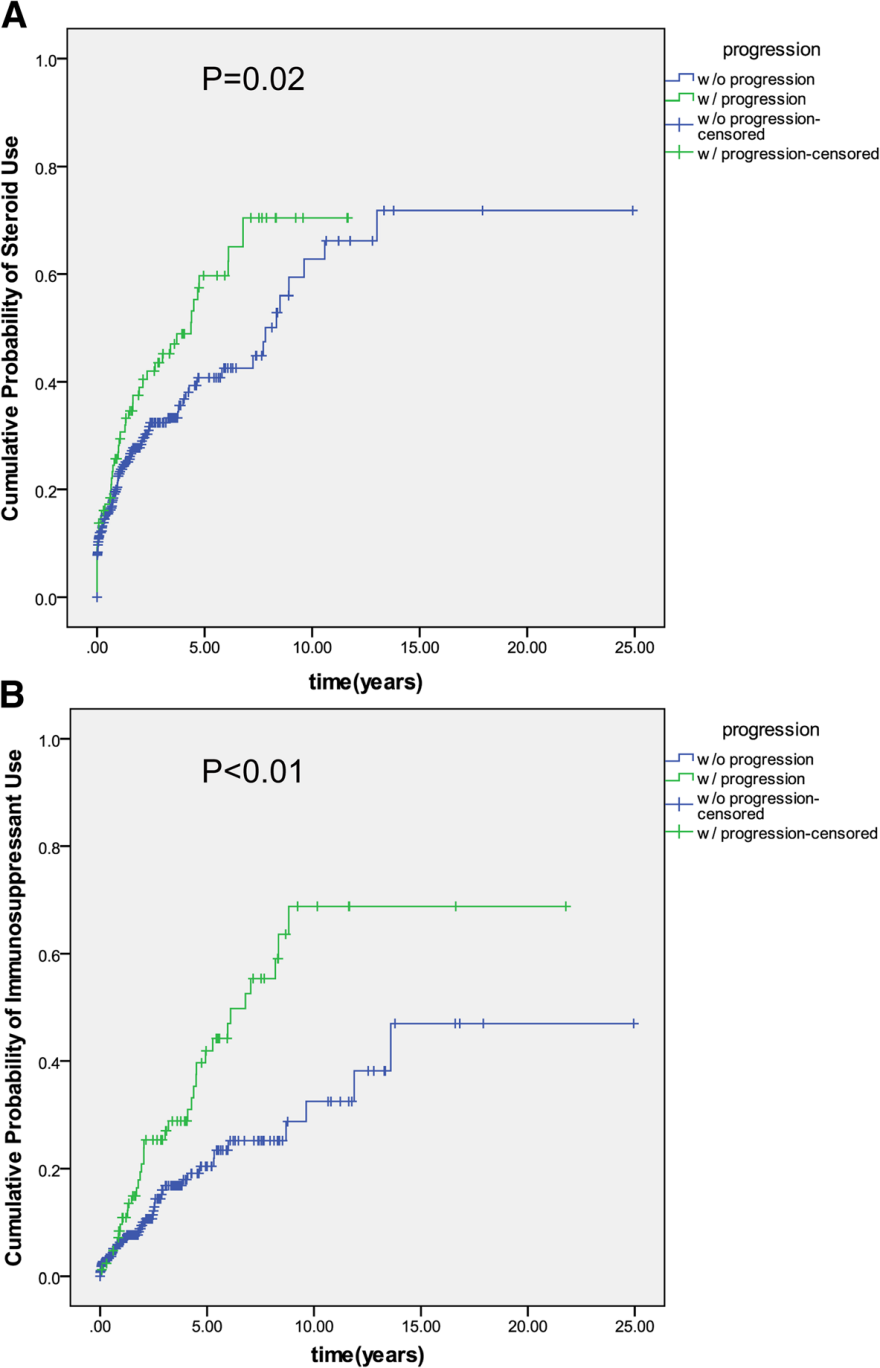
Cumulative rate of **a**) steroid utilization, and **b**) immunosuppressive agents use in patients with and without disease progression. Time to event was calculated from the date of index endoscopy evaluation to the date of steroids or IMM use or censoring (the last follow-up). Kaplan–Meier estimates were used to draw the cumulative incidence curves, compared by log-rank tests.

In terms of steroid utilization, 47 (47/91, 51.6%) patients with disease extension had used steroid, compared to 120 (120/427, 28.1%) patients without disease extension (*p* < 0.01,Table 3). The cumulative rate of steroid use in extenders was 28.2, 39.0, 43.6, 48.9 and 59.7% in the first 5-year post-diagnosis. In the non-extenders, the corresponding rates of steroid use were 22.9, 28.3, 32.4, 36.8 and 40.8%, respectively (*p* = 0.023, Fig. 4a). During the follow-up, 27 (27/91, 29.7%) of the 91 extenders were steroid -dependent compared with 36 (30.0%) of 120 patients without disease extension. The incidence of steroid -dependence were 6.5, 35.1, 43.3, 46.3 and 52.3% in the group of patients with disease extension during the first 5-year post-diagnosis, compared to that of 12.6, 21.0, 29.3, 31.2 and 36.2% in patients without disease extension (*p* = 0.08).

**Table 3:** The outcomes between patients with and without disease extension during follow-up

	extenders (*n* = 91)	non-extenders (*n* = 427)	*P* value
Steroid use	47(51.6%)	120 (28.1%)	< 0.01
Immunosuppressive agents use	36 (39.6%)	48(11.2%)	< 0.01
Colectomy	2(2.2%)	5(1.2%)	0.98
Hospitalization	13(14.3%)	26(6.1%)	0.51
Severe UC	13(14.3%)	26(6.1%)	0.51

Note: Time to event was calculated from the date of latest endoscopy evaluation to the date of each event (steroids or IMM use etc.) or censoring (the last follow-up)

Similarly, 36(39.6%) of the 91 extenders initiated immunosuppressive agents compared to 48 patients (48/427, 11.2%) without disease extension (Table 3**)**. The cumulative rates of immunosuppressive agents use in patients with disease extension during the first 5 years after diagnosis were 9.6, 20.9, 27.0, 31.0, and 41.9%, compared to that of 6.3, 10.0, 16.0, 17.9 20.4% in patients without disease extension, respectively. As shown in Fig. 4b, the cumulative incidence of being immunosuppressive agents use was higher in extenders compared to non-extenders (*p* < 0.01).

Two of the 91(2.2%, 2/91) extenders underwent colonic resection, compared to 5 (1.2, 5/427) in 427 non-extenders. There was no significant difference in the cumulative rate of colectomy between the two groups (*p* = 0.98, Table 3).

Thirteen (13/91, 14.3%) of 91 extenders were hospitalized due to severely active UC compared to 26 (26/427, 6.1%) in non-extenders without significant difference (*p* = 0.51, Table 3).

## Discussion

To our knowledge, this study has the largest sample size to present disease extent progression in Asian patients with UC. We have shown that in a Chinese cohort of UC cases, one in six UC patients experienced E1 to E2/E3 or E2 to E3 disease extension, and one in four patients with limited UC experienced disease extension to extensive colitis, after 7.5 years of follow-up. Only disease extent at diagnosis was identified as a clinical predictor for disease extension. Progression to extensive colitis resulted in a increased therapeutic requirements.

According to a recent retrospective study aiming to evaluate the natural disease course between pancolitis and non-pancolitis E3, pancolitis was associated with higher probabilities of cumulative relapse or hospitalization [18]. In the present study, we further classified E3 lesion based on hepatic flexure as well and found that, of patients diagnosed with E3, patients with pancolitis were associated with increased treatment requirements. Specifically, 38(33.3%) initiated steroids and 14(12.3%) initiated immunosuppressive agents in the 114 patients with pancolitis during the follow-up, whereas 16 (26.7%) initiated steroids and 6(10%) initiated immunosuppressive agents in the 60 patients with non-pancolitis E3.

Previous estimates of disease extension have varied between one-fifth to one-third of patients with E1 or E2 showed disease extension to E3 [6, 19, 20]. According to a recent meta-analysis, approximately one quarter of patients with limited UC demonstrated disease extension over time with most extension occurring during the first 10 years [21]. Disease extension may occur anytime after initial diagnosis with 31.1% within the first decade [22]. In our study, 91 (17.6%) had disease extension during a median follow-up of 7.5 years. The median time for any progression of disease extent was 16.1 months (IQR: 8.3–42.2 months). The cumulative rate of disease extension was 9.9, 14.9, 19.6, 24.6 and 30.5% at 1–5 year post-diagnosis, which was comparable to that of previous studies [3, 12].

In the present study, we found that only initial disease location had an impact on the risk of extension. Patients with initial E1/2 were at greater risk of extending to pancolitis. Several studies have demonstrated that patients with proctitis are at greater risk to extend to pancolitis [6]. In a recent study [20], the only predictor for UC extension was having E2 extent at baseline compared to E1. According to a recent meta-analysis, extension was 17.8% (95% CI 11.2–27.3) from E1 to E3, 27.5% (95% CI 7.6–45.6) from E2 to E3 and 20.8% (95% CI 11.4–26.8) from E1 to E2 [21]. In our study, though patients with E1 at baseline had the highest rates of disease extension of which the majority belongs to E1 to E2 extension, patients with E2 had a higher risk of progression to E3 compared to E1. This finding suggests that clinicians need to follow E2 patients more closely who are on higher risk for progression to pancolitis. Other previously reported risk factors for disease extension have also been examined. In the current study, there was a trend, albeit not statistically significant, toward increased risk of disease progression in patients diagnosed at a younger age. In contrast, smoking may have a protective effect against proximal disease extension [6]. Albeit there was a trend, we failed to find a statistically significant difference between smokers and non-smokers (*p* = 0.171). Altogether, risk factors have been largely inconsistent across studies and better predictors of disease progression are needed.

We also found that UC disease extension was associated with increased therapeutic requirements. Prior studies have examined the relationship between disease extension and subsequent outcomes [3, 5, 6, 10, 22]. Patients with disease extension tent to have a poor prognosis [6, 10]. For example, proximal progression was preceded by a flare-up in the majority of patients with UC according to a retrospective study [22]. In the population-based IBSEN cohort, there cumulative rates of colectomy were higher in extenders as compared to non-extenders [3]. Disease flare associated with progression also follows a refractory course with higher therapeutic needs [5]. In a Danish population-based inception cohort study, patients with proximal disease progression were more likely to be steroid-refractory, with greater need of immunotherapy, hospitalization and colectomy, as compared to non-extenders [20]. In the present study, UC extension resulted in poorer outcomes evidenced by increased risk of flare-ups, increased need for steroids and immunosuppressive agents, and higher rate of steroid-dependence.

In the current study, we failed to demonstrate a direct causal relationship between disease extension and increasing colectomy rates probably due to the relative low rate of colectomy (1.4%). Although our study was conducted in a referral center, the colectomy rate appears to be comparable with that of previous Eastern population-based studies [23, 24]. Previous studies have suggested that Chinese UC patients tent to have a milder disease course as compared with Caucasians [12, 25]. Although the cause of this variation remains unclear, possible factors included less extensive disease, better response to medical therapy, and less acceptance of colectomy by patients and/or physicians [26]. Another potential explanation might be that disease extent per se maybe not really a dictating factor for colectomy and it is more about other predictors ie, underlying severity of inflammation and/or refractoriness to medical treatments. Nonetheless, further studies are warranted to elucidate the correlation between disease extension and colectomy.

There were several limitations to our current study. First, our findings should be interpreted carefully due to the retrospective and referral center–based design of our investigation. Further long-term population-based study are required in order to reduce the selection bias. Second, due to the relative low rate of colectomy, we failed to find a relationship between disease extension and increased chance of colectomy. Last but not least, it is unknown whether any treatments or interventions decrease the risk of disease extension. According to our study, none of the medical treatment during follow-up associated with reduced disease extension, future research should investigate treatments or interventions that could prevent disease progression from E1/E2 to E3.

## Conclusions

UC is a dynamic disease and the proximal disease extension in the Chinese population was comparable to that in Caucasians. Proximal disease extension was associated with a more severe disease course with increased risk of treatment requirement. As the sole independent risk factor, our findings highlight the need of preventing progression in patients with limited UC.

## Abbreviations

5-ASA: 5-aminosalicylates
anti-TNF: Anti-tumor necrosis factor
CI: Confidence interval
CRP: C-reactive protein
CS: Corticosteroid
IBD: Inflammatory Bowel Disease
IQR: Interquartile range
UC: Ulcerative colitis
